# Parental Self-Compassion and Psychological Distress in Families of Children with Language and Speech Disorders: A Mixed-Methods Study in the Greek Context

**DOI:** 10.3390/children12111456

**Published:** 2025-10-26

**Authors:** Eirini Karakasidou, Anna Papadimitriou, Lida Triantafyllou

**Affiliations:** Department of Psychology, Panteion University of Social and Political Sciences, 17671 Athens, Greecetriantafyllou@ys.edu.gr (L.T.)

**Keywords:** self-compassion, psychological distress, parents, language disorders, speech disorders

## Abstract

Background/Objectives: Parents of children with language and speech disorders frequently face elevated psychological distress, which may hinder their caregiving capacity and overall well-being. In the Greek context, where research in this area remains limited, this study aimed to explore the relationship between parental self-compassion and psychological distress (depression, anxiety, and stress) among families of children diagnosed with such disorders. Methods: A mixed-methods design was employed. Quantitative data were collected from 150 parents (aged 27–55) using the Self-Compassion Scale (SCS) and the Depression Anxiety Stress Scales (DASS-21). Additionally, qualitative data were gathered through semi-structured interviews to gain deeper insight into parents’ emotional experiences, coping strategies, and support needs. Results: Quantitative analyses demonstrated a significant negative correlation between self-compassion and levels of depression, anxiety, and stress. The qualitative findings revealed themes of emotional burden, adaptive and maladaptive coping mechanisms, and the need for structured emotional support systems. Conclusions: The findings underscore the protective role of self-compassion in mitigating psychological distress among parents of children with communication disorders. Integrating self-compassion training and emotional support into family-centered intervention programs may enhance both parental well-being and child developmental outcomes.

## 1. Introduction

Parenting a child with Speech and Language Impairments brings a unique set of emotional and psychological strain far deeper than merely the burden of communication. For most parents, the journey starts with the shock of a diagnosis, followed by years of coping with therapists, school support services and the painful emotions of watching their child struggle to communicate and connect. This can be associated with considerable stress, emotional weariness and uncertainty about the future [1]. Parents in these situations often take on more than just the role of caretaker. They turn into emotional sounding boards, social go-betweens, and defenders of their kid’s interests. Children with speech and language impairments often experience social exclusion, problems in making friends and are even bullied, which further increases the emotional burden of the parent [1]. Parents need to be able to adjust to the development of their growing children, and that requires continuous emotional resilience and flexibility. Although clinical services (e.g., speech and language therapy) focus mainly on the child’s linguistic and cognitive outcomes, the parents’ psychological adjustment frequently remains unmet [2]. Nevertheless, there is evidence that parental mental health significantly impacts the effectiveness of conventionally implemented intervention and the development of the child [3,4]. Support of parents through their emotions promote parents’ continued involvement, ongoing demonstrations of healthy coping behaviors and collaboration with educators and healthcare professionals. In Greek culture, that is full of strong family ties and traditional caregiving roles, these battles can be amplified. Mothers and fathers, holding on to some cultural and societal expectations, frequently feel an enormous amount of pressure to place their child’s life above their own, leading to feelings of guilt, failure, and self-blame [5]. Resilience is assumed, if not always fostered. It is as if the emotional underpinning of their situation has not received any external validation; this can leave them feeling very alone, however calm and competent-seeming they are on the outside. Within this context, self-compassion—defined as being kind and understanding to oneself, feeling connected with others, and taking a balanced perspective when considering personal defects and suffering—may represent a key internal resource [6,7]. Unlike self-pity or self-indulgence, self-compassion means not making the thoughts and feelings in one’s head turn into actions that harm or kill, but self-compassion means responding to human suffering with kindness and understanding rather than with shame, criticism, or self-pity. Its practice has been in fact associated with decreased symptoms of anxiety, depression, and stress and increased use of adaptive emotional coping strategies [8,9]. Research on self-compassion in caregivers has burgeoned in recent years, with research on parents of children with autism and other neurodevelopmental conditions consistently suggesting the potential positive effects of self-compassion [10,11,12]. Nevertheless, research is scarce regarding parents of children with speech and language disorders, especially in Greece, where cultural expectations may affect the way that self-compassion is practiced or at least perceived. Moreover, current studies also tend to privilege quantitative data and do not make room for the subjective stories and emotional experiences of these parents. To contribute to filling these gaps, the present study investigated the association between self-compassion and psychological distress (depression, anxiety and stress) among a Greek sample of parents of children with language and speech disorders. Adopting a mixed-methods methodology, it brings to play the statistical robustness of quantitative assessment along with the richness of qualitative understandings. In this way, the study aims to guide the creation of more emotionally supportive interventions for caregivers—ones that both psychological evidence and personal experience would endorse.

### 1.1. Literature Review

According to Neff’s model of self-compassion [6], it comprises three correlated components, self-kindness, common humanity, and mindfulness. To work together, dissipate and shore up, these components create a buoyant, emotionally attuned attitude that enables one to move through one’s personal suffering with less self-reproach and more durability. Self-kindness is about being soothing and understanding toward oneself when feeling bad. A sense of shared humanity is the acknowledgement that imperfection and suffering are always a part of human experience. Mindfulness allows you to recognize painful thoughts and feelings while not getting stuck or overwhelmed by them.

Despite increasing popularity, self-compassion remains widely misunderstood. It can also be confused with self-pity—a feeling of being too identified with a negative feeling—or self-indulgence—taking the victim role in life and not being responsible for your actions. But true self-compassion is not either of these things. It is something meant to challenge itself and in turn inspire a new narrative, one that promotes honest self-reflection and personal evolution, not avoidance or complacency. One of the key blockades to self-compassion involves the fear that it would make one passive or not trying hard enough [7]. In reality, the reverse is true: self-compassion can enhance intrinsic motivation by allowing individuals to change out of kindness towards themselves as opposed to self-criticism.

For parents of children with speech and language impairments, the difference between self-caring and self-indulgence is particularly critical. These parents are forced to endure unrelenting frustration and demands on their emotions. Without the buffer of self-compassion, they become stuck in spirals of guilt, self-blame and emotional withdrawal. In contrast, those who treat themselves kindly may be better able to manage what is thrown at them as parents in a more patient and consistent manner.

Numerous researchers at this point have indicated the protective function of self-compassion in parents. For example, the parents of children with autism who reported higher levels of self-compassion had significantly less stress and depression [9]. These negative emotional states—such as anxiety, depression, and stress—have been widely studied in relation to self-compassion and well-being [10,11,13]. Moreover, in these studies, self-compassion was associated not only with positive psychological functioning, but also with more mindful parenting and emotional availability.

Caregiving situations for developmental disorder cases are usually long-term and emotionally demanding. Parents must balance children’s therapeutic needs with concerns about their functioning, independence and social integration in the long term. Under these circumstances, self-compassion is an important factor in determining how parents interpret and react to their daily hassles. Neff and Germer [8] propose that self-compassion may lead to more acceptance-based coping strategies and a reduction in less helpful (or harmful) ones such as denial, avoidance, and self-criticism.

One of the potential consequences is compassion fatigue: emotional exhaustion, depersonalization, and reduced feelings of personal accomplishment among caregivers. First identified amongst healthcare staff, compassion fatigue is being acknowledged in parental caregivers [14,15]. As parents distance themselves emotionally to protect themselves, children can experience this distance as emotional neglect or disconnect, which adds another layer of difficulty to their lives through language or speech difficulties.

Researchers found that self-compassion can protect against burnout. For example, there is a negative relation between emotional exhaustion and self-compassion [16]. More recent studies have supported these results in parents of children with chronic care conditions [17]. These parents also reported less burnout, as well as increased emotional regulation and parenting cohesion.

In a similar way, severity of the child’s disorder has been associated with stress in parents of children with communication disorders [18]. Yet this is tempered by self-compassion—where higher self-compassionate parents report less stress, even when confronted with relatively severe difficulties [12,19]. This result was confirmed by a recent meta-analysis [20] which reported that self-compassion was strongly related to lower levels of anxiety and depression and higher well-being in caregivers of children diagnosed with different neurodevelopmental disorders.

The potential of self-compassion has prompted researchers to investigate intervention type approaches. The Mindful Self-Compassion (MSC) program has been indicated to significantly decrease psychological stress across several populations [21], such as parents to children with disabilities. Compassion-focused therapy (CFT), an evidence-base therapeutic model developed by Gilbert [22], has also shown beneficial effects on shame, self-criticism and emotional well-being. While these programs have largely been examined in parents of children with autism or intellectual disability, preliminary results even indicate a potential for efficacy in families with speech–language disorders [23,24].

It should be noted that, in most of the intervention studies applying the AT, just mothers have been included, while fathers and other caregivers have not been considered [25]. Furthermore, the population of caregivers for children with Language and Reading Neurodevelopmental Disorders (LARND) have not been the focus in previous studies, even if their negative caring experience is expected at least to be compared with parents of children with other developmental disorders. Furthermore, much of the existing research uses only quantitative methods, missing the context of a very personal and emotional expression that qualitative data can give.

Finally, cultural issues sometimes fail to be addressed in these studies. In a global parenting context, and especially in the southern European context, such as that in Greece, influenced by parenting norms that value self-sacrifice, toughness and perfectionism—particularly among mothers—such an attitude of self-compassion might seem unintuitive and even unacceptable. The development and adaptation of self-compassion programs with respect to cultural aspects as mindfulness values represent an initial step for making such programs more accessible and effective.

This study aims to fill these gaps by focusing on Greek parents of children with speech and language disorders and by applying a mixed-method design that embraces not only statistical connections but also lived emotional experiences. In this way, it expands the larger discourse on ways that self-compassion provides benefit to the mental health and caregiving ability of this neglected population.

### 1.2. Aim of the Study

This study seeks to explore self-compassion levels among parents of children diagnosed with speech and language disorders in Greece. It also aims to investigate how self-compassion relates to psychological well-being, particularly in terms of depression, anxiety, and stress symptoms. By integrating both quantitative and qualitative approaches, the research attempts to deepen our understanding of the emotional experiences of caregiving.

### 1.3. Research Hypotheses

Parents who report higher levels of self-compassion will exhibit significantly lower symptoms of depression, anxiety, and stress, as assessed by the DASS-21 scale.

Those with greater self-compassion are expected to demonstrate healthier emotional coping mechanisms and reduced tendencies toward self-criticism, as identified through in-depth interviews.

Insights from the qualitative data will complement and enrich the quantitative results by highlighting how self-compassion supports emotional resilience in the daily challenges of parenting a child with communication difficulties.

## 2. Methodology

### 2.1. Research Design

This study followed a mixed-methods approach, combining both quantitative and qualitative data to explore the relationship between parental self-compassion and psychological distress in a more nuanced way. The rationale for using this approach was to not only measure patterns and correlations statistically but also to hear directly from parents about their lived experiences, something numbers alone cannot fully capture.

A convergent parallel design was used, meaning both types of data were collected at the same time but analyzed separately. The integration of results happened during interpretation, allowing us to compare findings from both strands. This helped to ground statistical trends in real-world experience and brought personal insight into the empirical findings.

### 2.2. Participants

A total of 150 parents participated in the study, ranging in age from 27 to 55 years. All were primary caregivers of children aged 3 to 12 who had been formally diagnosed with speech or language disorders by certified speech-language pathologists. The majority of participants identified as mothers (78%), while 22% were fathers. In terms of educational background, 38% had completed secondary education, 44% held a university degree, and 18% had pursued postgraduate studies. Socioeconomic status varied across the sample, with approximately 40% reporting low to moderate household income, 45% middle-income, and 15% high-income levels, reflecting a diverse caregiving population.

Participants were recruited through a diverse range of sources, including therapy clinics, special education institutions, and organized parent support groups, across both urban and semi-urban regions of Greece. Recruitment was facilitated via email invitations and QR codes distributed at various events led by the research team. This multi-channel recruitment strategy aimed to capture a broad range of caregiving experiences and enhance the representativeness of the sample in terms of geographic and service access diversity. As is common in caregiving studies, most of the participants (*n* = 104) were mothers, reflecting broader trends in parental involvement. Importantly, the group represented a mix of educational and socioeconomic backgrounds, which allowed for a broader look at how different life circumstances might shape psychological experiences.

The sample size of 150 participants was determined based on recommendations for detecting medium effect sizes (f^2^ = 0.15) with sufficient statistical power (0.80) in regression analyses involving multiple predictors [26]. This number was also consistent with prior empirical studies examining psychological constructs among parent caregivers, allowing for meaningful subgroup comparisons and the inclusion of relevant covariates.

To deepen the qualitative component of the study, a purposive subsample of 15 parents (12 mothers and 3 fathers) was selected from the larger participant pool. These individuals were all primary caregivers of children aged 3 to 12 diagnosed with speech or language disorders. The sampling strategy followed a maximum variation logic, aiming to include participants from diverse gender, educational, and socioeconomic backgrounds, as well as from different geographic regions. This approach was intended to ensure a heterogeneous range of caregiving perspectives, while maintaining coherence around the shared experience of raising a child with communication difficulties. Recruitment continued until thematic saturation was reached, at which point no new themes emerged during interview analysis.

### 2.3. Instruments

#### Quantitative Measures

Two validated questionnaires were used:

The Self-Compassion Scale (SCS), developed by Neff [6] and adapted into Greek by Karakasidou and her colleagues [27], was used to assess how compassionate participants were toward themselves. This 26-item scale consists of six subscales: Self-Kindness, Self-Judgment, Common Humanity, Isolation, Mindfulness, and Over-Identification. Respondents rate their agreement with each item on a 5-point Likert scale ranging from 1 (almost never) to 5 (almost always). Higher scores indicate greater levels of self-compassion. The Greek version has shown high internal consistency and construct validity, supporting its use in diverse populations. In the present study, Cronbach’s alpha coefficient for the total SCS was 0.89, indicating excellent internal reliability.

The Depression Anxiety Stress Scales (DASS-21), validated for use in Greece [28] measured the extent of psychological distress across three key domains: depression, anxiety, and stress. This 21-item short-form version includes 7 items per subscale, also rated on a 4-point Likert scale (0 = did not apply to me at all, 3 = applied to me very much or most of the time). DASS-21 is widely used in clinical and community samples and is sensitive to variations in emotional distress. The Greek adaptation has demonstrated robust psychometric properties. In the current sample, Cronbach’s alpha coefficients were a = 0.86 for Depression, a = 0.84 for Anxiety, and a = 0.88 for Stress, reflecting high internal consistency.

### 2.4. Qualitative Sampling and Interview Procedure

To explore the emotional experiences of parents caring for children with communication difficulties, in-depth, individual semi-structured interviews were conducted with 15 purposively selected participants. This method enabled a guided yet flexible discussion, encouraging participants to articulate personal experiences while addressing core themes such as emotional regulation, coping strategies, and perceived support. Semi-structured interviews are well-suited for this kind of inquiry, offering both structure and depth [29].

Interviews were conducted either face-to-face or via secure video conferencing, depending on participant preference and location. Each session lasted approximately 30 to 45 min and was conducted in Greek, the participants’ native language, to foster comfort and authentic expression. A flexible interview guide ensured consistency while allowing participants to elaborate on topics of personal relevance. Sample prompts included:

- “What do you say to yourself when you are feeling overwhelmed?”
- “Tell us about a time when you felt strong or supported as a parent.”
- “What do you believe professionals sometimes fail to understand about your emotional experience?”

All interviews were audio-recorded and transcribed verbatim with participants’ informed consent. Ethical approval was granted by the Research Ethics Committee of Panteion University (56/15 July 2025). Transcripts were anonymized, and data were securely stored in accordance with ethical standards.

Thematic analysis was conducted following the six-phase framework developed by Braun and Clarke [29], which provides a systematic yet flexible approach to identifying, analyzing, and reporting patterns (themes) within qualitative data. This method is particularly well-suited to exploring rich, subjective experiences, such as those involved in caregiving, and allows for the integration of both semantic and latent content [30]. Specifically, the analysis involved (1) familiarization with the data through repeated reading of transcripts; (2) generation of initial codes using an inductive, data-driven approach; (3) searching for themes by collating codes into potential overarching patterns; (4) reviewing themes to ensure internal coherence and consistency with the coded extracts and the entire dataset; (5) defining and naming themes to capture their essence; and (6) producing the final report, which involved selecting vivid, representative quotations to illustrate key findings. This qualitative component was designed to complement the quantitative results by illuminating the emotional nuances and lived experiences of caregiving that may be overlooked in survey-based research.

### 2.5. Data Analysis

#### 2.5.1. Quantitative Analysis

Quantitative data were analyzed using SPSS (Version 26). Basic statistics were used to describe trends, followed by Pearson correlations to explore how self-compassion related to depression, anxiety, and stress. Then, multiple regression analyses were run to see whether self-compassion could predict psychological distress, even when accounting for variables like age, gender, and education level. Cohen’s d was calculated to assess the strength of these effects.

#### 2.5.2. Qualitative Analysis

Interview transcripts were analyzed using reflexive thematic analysis, following the six-phase framework outlined [29]. The analysis was inductive in nature, allowing themes to emerge organically from participants’ narratives rather than being guided by a pre-existing coding framework. The researchers adopted a constructivist epistemological stance, acknowledging that meaning is co-constructed between researcher and participant and shaped by context.

Coding was conducted manually. Two researchers independently coded all transcripts and then met to compare interpretations, discuss discrepancies, and reach consensus on code definitions and theme development. The process was iterative and collaborative, with continuous movement between data and analysis. Researcher reflexivity was maintained throughout by keeping analytic memos and engaging in regular team discussions about potential biases and positionality.

Additionally, member checking was conducted with a subset of participants (n = 4), who were invited to review and comment on preliminary themes to ensure the trustworthiness and resonance of the interpretations. This feedback helped refine some theme labels and confirm the relevance of the patterns identified.

Finally, integration of qualitative and quantitative findings occurred during interpretation. For instance, participants with lower self-compassion scores often described experiences marked by guilt and emotional strain, while those with higher scores reflected more balanced self-appraisal and emotional resilience. These cross-method patterns strengthened the coherence and validity of the conclusions drawn.

## 3. Quantitative Results: Correlation and Regression Analyses

### 3.1. Correlation Analysis

An initial normality test was conducted using the Kolmogorov–Smirnov criterion, which showed that the data followed a normal distribution (*p* > 0.05). Therefore, a parametric test was chosen. A Pearson correlation was conducted to examine the relationship between self-compassion and psychological distress, as measured by the total score on DASS-21. The analysis revealed a moderate, negative correlation (r = −0.61, *p* < 0.001). This result indicates that higher levels of self-compassion were associated with lower levels of psychological distress in this sample.

### 3.2. Regression Analysis

To further examine this relationship, a simple linear regression was performed with Self-Compassion Total Score as the predictor and DASS Total Score as the outcome variable(see Table 1). The model was statistically significant, F (1, 152) = 91.40, *p* < 0.001, b = −5.63 and explained approximately 37.6% of the variance in distress scores (R^2^ = 0.376).

Individually, when examining the specific dimensions of the DASS-21, a similarly negative relationship with self-compassion was observed with Depression (r = −0.52, *p* < 0.01), Anxiety (r = −0.49, *p* < 0.01) and Stress (r = −0.51, *p* < 0.01).

### 3.3. Thematic Analytical Findings

Three main themes emerged from the thematic analysis of the 15 semi-structured interviews, each of these reflecting a different aspect of the emotional terrain of the experience of parents of young children with speech and language disorders(see Table 2). These themes—Emotional Burden, Coping with Compassion, and Gaps in Services—are evidence of how the emotional dimension of the caregiving role is experienced, negotiated and challenged by carers.

#### 3.3.1. Theme 1: Emotional Burden

Most of the subjects claimed that solicitation had induced them to live through a great deal of emotional stress, guilt, fear, and self-blame. The parents often doubted that they were competent and worthy of credit for their child’s development issues. These feelings were made even worse by social shaming and the invisibility of their work.

Self-blame and a pervasive sense of guilt: Many parents felt responsible for their child’s diagnosis. Anxiety about the future included worried about long-term progress, peer acceptance, and academic achievement. “I can’t help but think it is somehow my fault—maybe I didn’t talk to him enough when he was little.” (Mother, age 38).

Social stigma and exclusion: Parents reported feeling judged or not understood by others like, “Sometimes people look at us like we’re doing something wrong—like it’s our fault he doesn’t speak ‘normally’. I’ve even had relatives say things like ‘maybe if you talked to him more, this wouldn’t have happened.” (Mother, age 39).

#### 3.3.2. Theme 2: Coping with Compassion

Mothers who reported greater self-compassion outlined more positive emotional regulation strategies. These women described that they practiced mindfulness, rephrased negative thoughts, and granted themselves emotional space not clouded by judgment.

Mindfulness practices: Breathing, awareness of self, and focus on the present moment were mentioned as techniques to help with relaxation. Cognitive restructuring: Individuals actively substituted negative internal comments with more encouraging statements. As a participant stated, “When I feel overwhelmed, I try to stop and breathe—just focus on the moment and not let my mind run away with fear.” (Father, age 42).

Emotional acceptance: Parents accepted their emotional difficulties rather than suppressing or denying these. As mentioned, “It wasn’t until I stopped beating myself up that I could be more present with my child.” (Father, age 41).

#### 3.3.3. Themes 3: Gaps in Services

Several recurrent concerns raised in the interviews, were the lack of emotional or psychological support for parents through healthcare and educational services. Though most felt their children had received good speech-language services, they highlighted a neglect of their own welfare in the two-tiered system.

Absence of caregiver-oriented mental health intervention: Participants bemoaned the unilateral approach of the programs. Parents expressed the need for programs that include both the child and the parent. “I used to think, ‘I’m failing as a mother.’ But now I try to tell myself, ‘You’re doing the best you can with what you know.” (Mother, age 36).

Peer support wanted: Some participants expressed a desire for support groups or parent networks. As a subject mentioned, “Everyone’s making such a big deal about my daughter’s progress—and yet no one ever asks me how I’m doing.” (Mother, age 35).

## 4. Discussion

This study provides preliminary evidence of an association between self-compassion and lower levels of psychological distress among parents of children with language and speech disorders. Quantitative findings demonstrated strong, consistent negative correlations between self-compassion and symptoms of depression, anxiety, and stress, as measured by DASS-21. However, due to the cross-sectional nature of the study, no causal claims can be made about the directionality of this relationship. These results align with a growing body of international research that identifies self-compassion as a protective psychological factor in caregiving populations [9,10,12].

Equally important were the insights gained from the qualitative interviews. Parents who scored lower on self-compassion often shared narratives marked by guilt, emotional exhaustion, and self-blame—feelings that appeared to intensify the emotional burden of caregiving. This finding is consistent with existing research indicating elevated levels of emotional exhaustion in parents of children with developmental disabilities [1,12]. In contrast, those with higher self-compassion described more balanced self-perceptions and healthier coping mechanisms, such as mindfulness and cognitive reframing. These parents expressed an ability to be kinder to themselves, which seemed to support their capacity to remain more emotionally present in their caregiving roles.

While the findings indicate a consistent association between higher self-compassion and lower psychological distress, the cross-sectional design of the study prevents any inference of causality. It remains unclear whether self-compassion reduces psychological distress or whether individuals experiencing lower distress are more likely to report greater self-compassion. Additionally, although the qualitative data included caregiver narratives that resembled emotional states such as burnout and compassion fatigue, these constructs were not directly assessed through validated instruments. Any discussion of them is therefore interpretive and should be understood as hypothetical rather than empirical. Future research using longitudinal designs and specific measures of caregiver strain could help to explore these relationships more rigorously.

“Coping with compassion” as the participants referred suggests that self-compassion may serve as a protective function in buffering emotional distress, which has been found amongst clinical and community caregivers [9,16]. The topic of the gaps in services points to an urgent requirement for family-centered interventional models to be meshed more effectively to services for communication disorders, reflecting similar signals in the literature of the need for broader systemic assistance [4,30].

This alignment between quantitative trends and qualitative themes adds credibility to the findings and underscores the value of a mixed-methods approach. While statistical measures allowed us to observe broad patterns, the personal narratives helped to explain how self-compassion operates in real life—how it shapes inner dialog, buffers emotional strain, and supports the development of sustainable caregiving practices. These qualitative results provide important information about the emotional experience of caregiving in families of children with communication difficulties. They not only confirm the quantitative findings (e.g., the protective function of self-compassion), but also highlight the pressing need for the development of integrated support packages that consider the mental health of caregivers as well as child-focused interventions.

In the Greek cultural context, these findings carry weight. Greek society places a high value on family responsibility, and caregiving—especially among mothers—is often associated with self-sacrifice and emotional endurance [5]. This can lead to internalized pressure to appear strong and always composed, even when experiencing emotional strain. As a result, self-compassion may be misinterpreted as selfishness or weakness, making it harder for caregivers to engage in emotionally supportive self-talk. Addressing this cultural stigma is essential if self-compassion interventions are to be accepted and effective within Greek families.

The findings also support theoretical models of self-compassion proposed by Neff [6] and Gilbert [22], who emphasize the importance of reducing self-judgment and increasing self-kindness to improve emotional well-being. These theories are especially relevant for parents managing ongoing caregiving demands, where long-term stress and uncertainty are common. Self-determination theory further supports this idea by highlighting how emotional support and a sense of agency enhance psychological functioning—elements that self-compassion naturally promotes.

Although burnout and compassion fatigue were not directly measured in this study, some participants described emotional states resembling these constructs. Moreover, our findings are in line with previous studies showing that self-compassion is associated with reduced burnout and compassion fatigue in caregivers [16,17]. These emotional states have been widely observed in caregivers of individuals with chronic conditions and may also be relevant for parents of children with speech and language disorders. As shown in prior research, when parents lack emotional support or coping tools, they may begin to emotionally withdraw from their caregiving roles, something that can negatively affect both their mental health and the child’s development [14,15].

Our study also echoes the findings of intervention-based research. Programs like Mindful Self-Compassion (MSC) have been shown to reduce stress, anxiety, and depression in various populations, including parents of children with developmental disabilities [21,31]. Other studies have shown that compassion-focused therapy (CFT) can increase well-being and reduce self-criticism in caregivers [23,24]. Although most of this work has focused on autism or intellectual disabilities, our results suggest that similar benefits could extend to families managing language and speech disorders.

From a clinical and educational standpoint, these findings have meaningful implications. They suggest that supporting caregiver well-being should be an integral part of therapeutic programs for children with communication disorders. Mental health screenings, psychoeducational resources, and structured self-compassion workshops could be implemented alongside speech-language interventions to provide a more holistic form of care.

To be most effective, such interventions must also be culturally sensitive. In Greece, for example, programs may need to address common misconceptions about self-care and provide language that aligns with local values. Normalizing emotional vulnerability and reframing self-compassion as a strength—not a weakness—will be essential in encouraging parents to engage with these practices.

Finally, the study supports a broader shift in the caregiving field, emphasizing that parental mental health is not secondary—but essential to sustainable care. By treating parents as individuals with emotional needs, and not just as facilitators of therapy, we can create more sustainable, empathetic care systems that support the entire family unit.

### Limitations and Future Research

While the present study offers valuable insights into the relationship between parental self-compassion and psychological distress, several limitations must be acknowledged. First, the sample may not fully represent the full range of socioeconomic diversity within the Greek population. Most participants were recruited from urban and suburban areas, where access to speech-language therapy and mental health services is relatively more available. Consequently, the experiences of parents in rural or economically under-resourced regions—who may face additional barriers to support—are likely under-represented. Future studies should aim to include a broader demographic spectrum to better understand how contextual factors shape parental well-being.

Second, the study relied on self-report measures, which are subject to social desirability bias and may not capture deeper, unconscious patterns of self-criticism or resilience. Although the inclusion of qualitative interviews helped to mitigate this limitation, observational and multi-informant data could further strengthen the validity of future research. Moreover, the qualitative component included a relatively small sample of 15 participants, which may limit the generalizability of the findings. Future studies would benefit from incorporating larger and more diverse qualitative samples to capture a broader range of caregiving experiences. Third, the cross-sectional design of this study limits the ability to draw causal conclusions about the influence of self-compassion on long-term mental health outcomes. While the results suggest a strong association between higher self-compassion and lower psychological distress, longitudinal research is necessary to examine whether self-compassion training has a sustained effect over time. Such studies could track changes in parental well-being before, during, and after targeted interventions, thereby informing the design of more effective support programs.

Another important consideration is the cultural specificity of self-compassion. Although the construction has been validated in multiple international contexts, its expression and perceived acceptability may vary across cultures. In Greece, where caregiving is often viewed through a lens of sacrifice, self-directed kindness may be misunderstood or undervalued. Future studies should explore culturally adapted self-compassion interventions that consider local values, family structures, and parenting expectations. Tailoring the language, delivery methods, and examples used in such programs could improve relevance and uptake.

Finally, additional research should investigate the influence of gender, education level, and caregiver role (e.g., mother, father, grandparent, or secondary caregiver) on both self-compassion and psychological distress. For instance, mothers may experience different emotional pressures than fathers due to societal expectations and traditional roles. Similarly, grandparents who serve as primary caregivers may face unique emotional and generational challenges that merit further exploration.

Additionally, self-compassion has been shown to serve as a protective factor against burnout and compassion fatigue in caregivers [16]. However, most of the existing research in this area has primarily focused on caregivers of individuals with dementia, as well as on nursing staff, medical personnel, and healthcare professionals. Given this, it would be highly meaningful for future studies to explore the protective role of self-compassion specifically in parents of children with language and speech disorders. These parents often face chronic emotional and practical demands that can lead to similar experiences of burnout and emotional exhaustion, even though these conditions may be presented in less obvious or socially acknowledged ways. Investigating self-compassion as a buffer in this context could shed light on how it helps prevent the gradual erosion of parental mental health processes that subtly, yet profoundly, impact both the parents and their children. Understanding and addressing these hidden vulnerabilities could lead to the development of more effective support systems and preventive interventions aimed at sustaining the emotional well-being of parents and, by extension, promoting healthier developmental trajectories for their children. Incorporating this line of inquiry into the broader field of caregiver research would not only fill a significant gap in the literature but also highlight the importance of psychological resilience and emotional self-care in families navigating the lifelong challenges associated with language and speech disorders.

Future research could also focus on implementing and evaluating self-compassion and compassion-focused therapy interventions specifically for parents of children with language and speech disorders. These interventions could help parents benefit more deeply from the cultivation of self-compassion and mindfulness, contributing positively to their mental health and overall well-being. There is a notable research gap in this area: while the effectiveness of such interventions has been studied primarily in parents of children on the autism spectrum, similar research in the context of language and speech disorders is significantly lacking. Investigating the efficacy of these interventions in this specific population would provide valuable new data on their impact and potential. Moreover, these interventions could be expanded to examine how parental self-compassion not only supports the parents’ mental health but also contributes to the development of interpersonal skills and other positive psychological traits in children with language and speech disorders. Since parents are the primary caregivers and role models for these children, their emotional well-being directly and indirectly influences their children’s development and adjustment. Understanding and supporting the psychological resilience of parents through targeted compassion-based interventions could, therefore, be a powerful pathway to enhancing both parent and child outcomes in the context of lifelong language and speech challenges.

Future research could benefit from adopting a more longitudinal perspective. Language and speech disorders are typically diagnosed at an early age—often during the preschool years—and they tend to persist throughout a child’s lifetime. These disorders are not “cured” in the traditional sense; rather, they evolve over time, with variations in symptomatology and manifestation depending on the child’s developmental and cognitive stages. Given this lifelong trajectory, it would be particularly meaningful for future studies to explore the development of self-compassion in parents of preschool-aged children diagnosed with language and speech disorders. Longitudinal research could then examine how self-compassion influences parental mental health and coping strategies over the course of the child’s development—from preschool through primary school, middle school, and high school. Such a long-term approach would not only shed light on the emotional and psychological resilience of parents but could also explore whether fostering self-compassion has a positive impact on the developmental outcomes and skill acquisition of children across different life stages. This might include examining whether children show differences in communication, academic progress, and social-emotional development depending on the emotional well-being and self-compassionate attitudes of their caregivers. In sum, integrating a developmental and temporal lens into future studies would enhance our understanding of how early interventions for parents—particularly those centered around self-compassion—can support both parental well-being and the adaptive functioning of children with language and speech disorders over time. By addressing these limitations and expanding the scope of inquiry, future research can more fully elucidate the protective and transformative potential of self-compassion in the lives of caregiving families.

## 5. Conclusions

In conclusion, self-compassion appears to play a protective role and contributes significantly to reducing anxiety, stress, and depression in parents of children with language and speech disorders. From the qualitative analysis of the interviews, it became evident that parents often experience a profound emotional burden. However, cultivating self-compassion helps them remain emotionally present in their children’s lives and better cope with the challenges they face. This inner resource enables them to adopt more adaptive strategies in their parenting and caregiving roles. Moreover, parents recognized the importance of having a supportive network, particularly within clinical practice settings. In their narratives, they frequently expressed feelings of guilt and admitted blaming themselves for their children’s difficulties. These self-critical patterns highlight the need for interventions that specifically target self-blame and internalized criticism. Compassion-focused interventions, such as CFT, have shown promise in addressing these issues. CFT was developed to alleviate deep-rooted feelings of shame—an area where many traditional therapeutic approaches fall short—thus filling a critical gap in psychological support. Such interventions not only help parents manage their own emotional distress but also indirectly benefit their children by fostering a healthier family dynamic. From the lived experiences of these parents, compassion supports them in adopting more constructive and emotionally sustainable strategies, ultimately enhancing both their well-being and that of their children. Importantly, parents pointed out the lack of adequate psychological support available to them, despite acknowledging how crucial such support is. This highlights the necessity of integrating parental support into the holistic therapeutic framework for children and adolescents with language and speech disorders. Therefore, it is highly beneficial that at the point of diagnosis, parents are referred to mental health professionals. Such referrals can help them manage the emotional burden, stress, anxiety, and depression they experience, as well as to cope with the persistent feelings of guilt and self-criticism. Furthermore, parents should be supported and encouraged to develop self-compassion as a means of effectively navigating the complex demands and challenges they encounter. While many existing studies [23,24] have focused exclusively on mothers, the present research included both mothers and fathers. This is a crucial strength, as the involvement of both parents is essential in the holistic treatment and support of children facing language and speech disorders. Including both caregivers ensures a more comprehensive approach and contributes to more balanced and effective outcomes for the entire family system.

## Figures and Tables

**Table 1:** Linear Regression Predicting Psychological Distress from Self-Compassion.

Predictor	Β	SE B	Β	t	P
Self-Compassion	−5.63	0.59	−0.61	−9.56	<0.001

Note. R^2^ = 0.376; Adjusted R^2^ = 0.371. Outcome variable: DASS-21 Total Score.

**Table 2:** Thematic analysis.

Themes	Subthemes	Frequency	Quotes from Thematic Analysis
Emotional Burden	Self-blame and a pervasive sense of guilt	14	“I keep wondering if there’s something I did or didn’t do that caused this…”
	Social stigma and exclusion		“People look at us like we’re doing something wrong… like it’s our fault.”
Coping with Compassion	Mindfulness practices	9	“When I feel overwhelmed, I stop and breathe—just focus on the moment.”
	Emotional acceptance		“It wasn’t until I stopped beating myself up that I could be more present with my child”
Gaps in Services	Absence of caregiver-oriented mental health intervention.	11	“Everyone’s focused on my daughter’s progress—but no one ever checks in on me.”
	Peer support wanted		“Everyone is focused on how my daughter is doing—but no one ever asks about me.”

## Data Availability

The data presented in this study is available on request from the corresponding author.
